# Nanopore-Based Surveillance of *Leishmania* Parasites in *Culicoides* Latrielle (Diptera: Ceratopogonidae) Caught from the Affected Community and Tham Phra Cave in Chiang Rai Province, the Endemic Area of Leishmaniasis in Northern Thailand

**DOI:** 10.3390/insects15050327

**Published:** 2024-05-02

**Authors:** Rinnara Ampol, Puckavadee Somwang, Pathamet Khositharattanakool, Chulaluk Promrangsee, Thanapat Pataradool, Piyapat Tepboonreung, Padet Siriyasatien, Kanok Preativatanyou

**Affiliations:** 1 Center of Excellence in Vector Biology and Vector-Borne Disease, Chulalongkorn University, Bangkok 10330, Thailand; rinnara.a94@gmail.com (R.A.); thanapat.p@chula.ac.th (T.P.); padet.s@chula.ac.th (P.S.); 2 Department of Parasitology, Faculty of Medicine, Chulalongkorn University, Bangkok 10330, Thailand; 3 Biomedical Technology Research Group for Vulnerable Populations, Mae Fah Luang University, Chiang Rai 57100, Thailand; puckavadee.som@mfu.ac.th (P.S.); pathamet.kho@mfu.ac.th (P.K.); 4 School of Medicine, Mae Fah Luang University, Chiang Rai 57100, Thailand; 5 Interdisciplinary Program of Biomedical Sciences, Graduate School, Chulalongkorn University, Bangkok 10330, Thailand; famezaflamingoclub@hotmail.com; 6 Medical Science Program, Faculty of Medicine, Chulalongkorn University, Bangkok 10330, Thailand; ptepboonrueng@gmail.com

**Keywords:** *Culicoides* biting midges, leishmaniasis, *Leishmania martiniquensis*, *Leishmania orientalis*, infection prevalence, genetic diversity, Northern Thailand

## Abstract

**Simple Summary:**

The number of leishmaniasis cases caused by autochthonous *Leishmania* (*Mundinia*) *martiniquensis* and *Leishmania* (*Mundinia*) *orientalis* has been continuously growing in Southeast Asia over the decades, particularly in Thailand. Recent evidence has suggested that *Culicoides* biting midges are the most likely natural vectors of autochthonous leishmaniasis in Thailand. Nevertheless, the epidemiology of vector infection and the genetic variation of *Leishmania* parasites in endemic areas have remained largely unknown. In this study, we report the high infection prevalence, sympatric circulation, and regional genetic diversity of two *Leishmania* (*Mundinia*) parasites in *Culicoides* spp. caught from the affected community and Tham Phra cave in Chiang Rai Province, Northern Thailand. These results provide us with a more complete understanding of the complexity of leishmaniasis transmission, which will be useful for establishing effective management and control measures for this neglected disease, especially in endemic areas of Northern Thailand.

**Abstract:**

In this research, we elucidated the species composition of *Culicoides* biting midges, infection prevalence, and genetic diversity of *Leishmania* parasites circulating in the affected community in Chiang Rai Province, being the most endemic area in Northern Thailand. A total of 146 parous and gravid females, belonging to at least twelve *Culicoides* species in five subgenera and one species group, were trapped from three collection sites with an overall *Leishmania* prevalence of 26.7% (39/146). *Leishmania* was detected, using *ITS1*-PCR, in *C. mahasarakamense* (15), *C. guttifer* (11), *C.* (*Trithecoides*) spp. (8), *C. jacobsoni* (2), *C. oxystoma* (2), and *C. orientalis* (1). The evidence of *Leishmania* infection in these last five species represents new records in Northern Thailand. Given a high infection rate in cavernicolous specimens, this indicates an increased risk of parasite exposure when visiting the cave. Using the nanopore amplicon sequencing, *L. martiniquensis* was ubiquitously identified in all positives, and more than half of these were also co-infected with *L. orientalis*. The genetic diversity analysis revealed 13 and 17 unique haplotypes for *L. martiniquensis* and *L. orientalis*, respectively. Higher haplotype diversity and relatively low nucleotide diversity were observed in both parasite populations, suggesting recent population divergence. Neutrality tests (Tajima’s *D* and Fu and Li’s *D*) showed to be significantly negative, indicating rapid population growth or a selective sweep. Moreover, dominant haplotypes of both *Leishmania* species were 100% identical to those in all leishmaniasis patients previously reported from Northern Thailand, strongly supporting the imperative role of *Culicoides* spp. in disease transmission. Essentially, this research provides the first entomological surveillance data representing the sympatric existence, transmission dynamics, and genetic complexity of two autochthonous *Leishmania* (*Mundinia*) parasites in several *Culicoides* species in the endemic area of Northern Thailand. This would contribute to a more complete understanding of the epidemiology of vector infection and facilitate the development of vector control programs to effectively reduce the transmission of this neglected tropical disease in endemic areas of Northern Thailand.

## 1. Introduction

Leishmaniasis is a neglected tropical disease caused by obligatory intracellular parasites belonging to the genus *Leishmania* [1]. This disease has traditionally been known to be transmitted by phlebotomine sand flies and is widely distributed across tropical and subtropical countries worldwide [1]. According to recent data from the World Health Organization, it is estimated that 50,000 to 90,000 new cases of visceral leishmaniasis and 600,000 to 1 million new cases of cutaneous leishmaniasis occur globally each year [2]. To date, over 50 *Leishmania* species have been described, and 20 of these can cause disease in humans [2]. These parasites have been taxonomically classified into four subgenera, namely *Leishmania*, *Viannia*, *Sauroleishmania*, and the recently described *Mundinia* (previously known as ‘*L. enriettii* complex’) [3]. There are six member species in the new subgenus *Mundinia*, consisting of *L. enriettii* [4], *L. martiniquensis* [5], *L. orientalis* [6], *L. chancei* [7,8], *L. procaviensis* [8], and *L. macropodum* [9,10].

Since 1996, leishmaniasis has been considered an important public health issue in Thailand due to the continuously increasing incidence of autochthonous cases and asymptomatic people, especially in the northern and southern provinces of the country [11]. Currently, this emerging disease has been proven to be caused by two *Leishmania* species of the newly classified subgenus *Mundinia*, namely *L. martiniquensis* and *L. orientalis*, as mentioned earlier [5,6,11,12,13]. As previously recorded, most of the autochthonous cases in Thailand were diagnosed with *L. martiniquensis* [11,12]. Clinical leishmaniasis cases due to *L. martiniquensis* usually present with visceral leishmaniasis; however, cutaneous or mucocutaneous leishmaniasis can also develop concurrently, particularly in individuals with immunocompromised status [5,12]. On the contrary, cases of *L. orientalis* have sporadically been reported, typically manifesting as localized cutaneous leishmaniasis [6,13]. The rising prevalence of this disease has therefore emphasized the importance of transmission interruption by vector population control, especially in endemic areas. However, the information on proven natural vectors and parasite reservoirs of these two *Mundinia* species remains unclear.

Phlebotomine sand flies have formerly been implicated as natural vectors of *Leishmania* in the subgenera *Leishmania*, *Viannia*, and *Sauroleishmania* across several geographic locations [14]. In Thailand, more than 36 sand fly species belonging to five genera—namely *Sergentomyia*, *Phlebotomus*, *Idiophlebotomus*, *Grassomyia*, and *Chinius*—have been recorded in several localities throughout Thailand [15,16,17,18]. Molecular detection of these two *Mundinia* species has previously been reported in several Thai sand fly species—namely *Se. khawi* [19], *Se. barraudi* [20], *Se. iyengari* [21], *Gr. indica* [18], and *Ph. stantoni* [22]—collected from endemic areas. It could be explained by the fact that *Leishmania* parasites may survive inside the peritrophic matrix for several days after acquiring infectious blood meals, possibly resulting in positive PCR results [23]. However, recent experimental studies revealed that several *Mundinia* species could not develop or poorly develop late-stage infection in the midgut of phlebotomine sand flies, implying that sand flies might not be the main natural vectors of *Leishmania* parasites in this subgenus [23,24,25]. In contrast, all *Mundinia* species could successfully colonize the stomodeal valve and complete the full development into the infective metacyclic stage in *C. sonorensis*, supporting vector competence and the putative role of *Culicoides* species in parasite transmission in nature [23,24,25]. It is therefore highly plausible that this non-phlebotomine insect group acts as the primary vector responsible for the transmission of these parasites, particularly in the endemic areas of Thailand.

*Culicoides* biting midges are globally widespread hematophagous insects classified in the family Ceratopogonidae, order Diptera. More than 168 species have been recorded from different localities of Southeast Asian countries [26]. With veterinary importance, this insect genus has previously been known as the biological vector of several animal arboviruses, including the African horse sickness virus, bluetongue virus, epizootic hemorrhagic disease virus, and Schmallenberg virus [27]. Interestingly, several *Leishmania* species of the other subgenera, including *L*. (*Leishmania*) *amazonensis* [28], *L*. (*L*.) *infantum* [29], *L*. (*L*.) *mexicana* [30], and *L*. (*Viannia*) *braziliensis* [28], have also been molecularly detected in *Culicoides* specimens collected from diverse geographic origins, suggesting the probable involvement of biting midges in leishmaniasis transmission.

Although *Culicoides* biting midges are common and have a high species richness throughout the country, the number and relative abundance of *Culicoides* species, which are thought to be the primary vectors of autochthonous leishmaniasis, especially in endemic areas of Thailand, remain largely unknown. Thus far, only a single species—namely *C. mahasarakhamense,* collected from the vicinity of a leishmaniasis patient’s house in Lamphun Province—has been recorded as a putative vector of autochthonous *L. martiniquensis* in Northern Thailand [31]. Accordingly, investigations into *Culicoides* fauna with monitoring of infection prevalence are essentially required to better understand the complexity of leishmaniasis transmission and enable the effective management and control of this emerging disease, particularly in highly endemic areas of Northern Thailand.

In the present research, we aimed to explore the species diversity of *Culicoides* biting midges, infection prevalence, and sympatric circulation of *Leishmania* parasites in three localities located in Chiang Rai Province, most affected by leishmaniasis in Northern Thailand. Here, we applied an *ITS1*-based metabarcoding strategy using the nanopore sequencing platform to generate high-throughput data with high taxonomic resolution. Furthermore, the variant identification from metabarcoding data will help us better comprehend the regional genetic diversity of autochthonous *Leishmania* parasites circulating in the investigation areas in this study.

## 2. Materials and Methods

### 2.1. Ethics Statement

The research methodology for entomological investigation and molecular analysis was approved by the animal research ethics committee of Chulalongkorn University Animal Care and Use Protocol (CU-ACUP), Faculty of Medicine, Chulalongkorn University, Bangkok, Thailand (COA No. 008/2566).

### 2.2. Investigation Areas, Biting Midge Collection, and Morphological Identification

**Figure 1:**
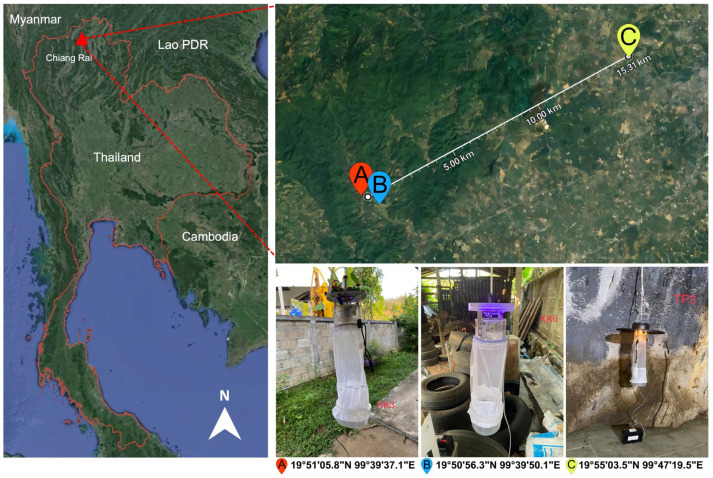
The location and geographic coordinates of *Culicoides* trapping sites in Mae Kon and Mae Yao Subdistricts, Muang District, Chiang Rai Province, Northern Thailand. The light traps were installed within the patient’s residential area (A), near the grocery shop (B), and within the hall of Tham Phra cave (C). The satellite image was modified from the Google Earth website (https://earth.google.com/web/search/Thailand (accessed on 15 July 2023)).

Insect collection was conducted in July 2023 in Ban Pang Rim Kon village, including the residence of a patient recently diagnosed with multiform leishmaniasis (19°51′05.8″ N, 99°39′37.1″ E) and a nearby grocery shop (19°50′56.3″ N, 99°39′50.1″ E) in Mae Kon Subdistrict, and Tham Phra cave (19°55′03.5″ N, 99°47′19.5″ E) located in Mae Yao Subdistrict, Muang District, Chiang Rai Province, Northern Thailand, as depicted in Figure 1. The distance between the village and Tham Phra cave was approximately 15.3 km. The light traps were installed approximately one meter above the ground at different places and operated from dusk to dawn for three consecutive days. All insect specimens collected the next day would be immobilized by being knocked out in the freezer for 30 min and then inspected under a stereomicroscope (EZ4 HD, Leica, Germany) to sort female *Culicoides* individuals from males and other insect species, according to distinct morphological structures, i.e., non-plumose antennae and wing venation patterns. In this study, nulliparous females, which generally predominated the traps and had never fed on a blood meal, were discarded from the female collection. The presence of burgundy-red pigment within the abdominal wall of parous midges is typically used to distinguish between the nulliparous and parous *Culicoides* [32]. Only parous, gravid, and blood-engorged ones, which had entered the gonotrophic cycle, would be included for analysis. All midge specimens were then morphologically identified according to the illustrated keys to *Culicoides* species of Southeast Asia described by Wirth and Hubert [26] and the formal descriptions of *C. mahasarakhamense* by Pramual et al. [33]. Then, the midge specimens were kept dry at −80 °C before downstream genomic DNA isolation.

### 2.3. Genomic DNA Isolation from Culicoides Samples

To preserve specimens, each *Culicoides* individual was extracted for total genomic DNA (gDNA) without crushing or homogenization using a non-destructive protocol previously described by Santos et al. [34] Concisely, each specimen was submerged for digestion in 200 µL of lysis buffer containing Proteinase K and incubated at 50 °C overnight. The lysate was subsequently purified using the GenElute™ Mammalian Genomic DNA Miniprep Kit (Sigma-Aldrich, St. Louis, MO, USA). The DNA concentration and quality were evaluated using the NanoDrop 2000c spectrophotometer (Thermo Fisher Scientific, Waltham, MA, USA). The obtained gDNA specimens were preserved at −20 °C for long-term storage until use. After extraction, the insect remains were kept in 70% ethanol solution at room temperature for further species confirmation.

### 2.4. Molecular Screening of Leishmania DNA by ITS1-PCR

The gDNA of parous and gravid specimens was used as a template for PCR-based detection, universally targeting the internal transcribed spacer 1 (*ITS1*) region of *Leishmania* species. The *ITS1*-specific PCR reactions were performed using the forward and reverse primers as follows: LITSR2 (5′-CTGGATCATTTTCCGATGATT-3′) and L5.8S inner (5′-GTTATGTGAGCCGTTATCC-3′) [35] to amplify a PCR product with an approximate size of 272–280 bp, encompassing the *ITS1* region. The PCR components were prepared in a total volume of 50 µL mixture containing 4 µL of extracted gDNA, 1.5 µL of 10 µM each primer, 25 µL of 2X KAPA HiFi HotStart ReadyMix (Roche, Basel, Switzerland), and 18 µL of nuclease-free water. The PCR thermal condition included initial denaturation at 95 °C, 5 min; 35 cycles of denaturation at 98 °C, 30 s, annealing at 53 °C, 30 s, extension at 72 °C, 30 s; and final extension at 72 °C, 10 min. The gDNA extracted from cultured *L. martiniquensis* promastigotes (WHO code: MHOM/TH/2012/CULE1) was used as a positive control. The amplification products were verified on 1.5% (*w*/*v*) agarose gel electrophoresis stained with ethidium bromide and then visualized by the GelDoc Go Imaging System (Bio-Rad, Hercules, CA, USA).

### 2.5. MinION^®^ Amplicon Sequencing and ITS1 Consensus Calling

Using the MinION^®^ nanopore sequencing technology, the positive amplicons were also sequenced to identify parasite species and demonstrate the multiple *Leishmania* co-infections in positive *Culicoides* samples. The Native Barcoding Kit 96 V14 (cat. no. SQK-NBD114.96, Oxford Nanopore Technologies, Didcot, UK) was deployed for barcoded DNA library preparation following the manufacturer’s protocol. For multiplexing, each sample containing 200 fmol of barcoded amplicons was pooled together and then ligated with the sequencing adapter. After clean-up, 20 fmol of the final prepared library was loaded onto the MinION^®^ R10.4.1 flow cell for 5 h. Super-accuracy base calling, demultiplexing, and adapter-barcode trimming were performed using the Dorado version 0.5.3 with the latest model dna_r10.4.1_e8.2_400bps_sup@v.4.3.0. Chopper version 0.7.0 [36] was applied to filter out low-quality reads with Q-scores lower than 20. Only reads with a length of 250–320 bp were to be processed for downstream analysis. Filtered reads were sorted based on their similarity in sequence and length via amplicon_sorter.py (version 2024_02_20) [37] and then error-polished with raw reads using Medaka version 1.11.3 to generate all accurate consensus sequences for each positive sample.

### 2.6. BLASTn Alignment and Phylogenetic Analysis of ITS1 Consensus Sequences

For the taxonomic assignment, the obtained consensus *ITS1* sequences were aligned against the GenBank references using the nucleotide Basic Local Alignment Search Tool (BLASTn) (http://blast.ncbi.nlm.nih.gov/Blast.cgi, accessed on 6 October 2023). To perform phylogenetic analysis, the *ITS1* sequences obtained in this study along with 39 sequences from GenBank representing different hosts and different geographic origins were aligned and analyzed by the MEGA X [38] software using the maximum likelihood method with Kimura 2-parameter model with discrete gamma distribution (K2+G). The bootstrap testing was calculated with 1000 replicates.

### 2.7. Haplotype Network Analysis

Genetic variability of the *Leishmania ITS1* sequences detected in *Culicoides* in this study and those from leishmaniasis patients previously reported from the leishmaniasis areas in Northern Thailand was evaluated by haplotype network analysis based on single-nucleotide variation (SNV) and small insertions and deletions (indels). A randomized minimum-spanning network was generated using the package ‘pegas’ version 1.3 [39] in RStudio version 2023.12.1+402 [40] to visualize the active haplotypes. Genetic diversity indices including the number of haplotypes (H), number of polymorphic sites (S), the average number of nucleotide differences (k), haplotype diversity (Hd), nucleotide diversity (π), and Tajima’s *D* statistics were calculated using the package ‘pegas’ version 1.3. Fu, and Li’s *D* statistics were also estimated using the package ‘PopGenome’ version 2.6.1 [41]. A *p*-value less than 0.05 was determined to be statistically significant.

## 3. Results

### 3.1. Species Composition and Relative Abundance of Culicoides Species

**Figure 2:**
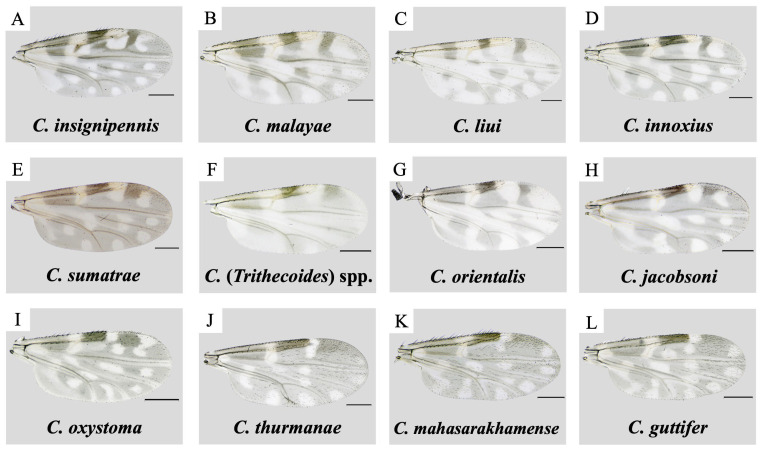
Wing morphology and pigmentation patterns of *Culicoides* species belonging to five subgenera and one species group trapped in this study. *Culicoides* subgenera or species group were represented as follows: *Hoffmania* (**A**–**E**); *Trithecoides* (**F**); *Avaritia* (**G**,**H**); *Remmia* (**I**); *Shermani* group (**J**); and *Meijerehelea* (**K**,**L**). The scale bar represents 200 µM.

A total of 146 non-engorged parous and gravid female *Culicoides* biting midges were captured from three collection sites. These comprised seventy-one and six from the vicinity of the patient’s house and the nearby grocery store, respectively, in Mae Kon Subdistrict, and sixty-nine from Tham Phra cave in Mae Yao Subdistrict. No blood-fed females were trapped in these three locations. Nulliparous females which dominate the traps were not included in the downstream analysis. Parous and gravid *Culicoides* specimens were then morphologically identified as at least twelve distinct species belonging to five subgenera, namely *Hoffmania*, *Trithecoides*, *Avaritia*, *Remmia*, *Meijerehelea*, and *Shermani* group, according to the characteristic wing pigmentation patterns, as illustrated in Figure 2. *Meijerehelea* is the subgenus with the broadest distribution across three trapping sites in this study. In the vicinity of the patient’s residence, the collection exhibited the highest species richness (*n* = 11), with *C.* (*Trithecoides*) spp. (*n* = 18/71, 25.4%), *C. insignipennis* (*n* = 15/71, 21.1%), and *C. orientalis* (*n* = 12/71, 16.9%) as the three most predominant species. Only three samples of *C. oxystoma*, two of *C. jacobsoni*, and a singleton of *C. mahasarakhamense* were captured near the grocery shop. For Tham Phra cave, *C. guttifer* presented the highest number of specimens (*n* = 41/69, 59.4%), followed by *C. mahasarakhamense* (*n* = 28/69, 40.6%). The information on species composition and relative abundance of collected midges is summarized in Table 1.

### 3.2. Prevalence of Leishmania Infection in Culicoides and ITS1 Amplicon Sequencing

*Leishmania* was molecularly detected in 39 out of 146 specimens obtained from three collection sites, using *Leishmania ITS1*-specific PCR. The overall infection prevalence was 26.7%. Within the patient’s residence, out of 71 specimens, twelve, including *C.* (*Trithecoides*) spp. (8), *C. jacobsoni* (2), *C. orientalis* (1), and *C. mahasarakhamense* (1), tested positive for *Leishmania,* with a local prevalence of 16.9%. The positive detection was also found in two samples of *C. oxystoma* and a singleton of *C. mahasarakhamense* collected near the grocery store. Furthermore, a higher prevalence (*n* = 24/69, 34.8%) was observed in eleven and thirteen specimens of *C. guttifer* and *C. mahasarakhamense*, respectively, caught from Tham Phra cave.

We applied a metagenomic approach using MinION^®^ nanopore sequencing which would provide us with high-throughput data to evaluate multiple *Leishmania* infections and enable variant identification. After filtering low-quality and unclassified reads out, 639,648 high-quality sequenced reads from all positive samples were clustered and error-polished to generate highly accurate consensus sequences. A total of 67 *ITS1* consensus sequences were identified, as shown in Table S1, with one to four consensus variants produced for each sample.

### 3.3. Molecular Identification and Phylogenetic Confirmation of Leishmania Species

**Figure 3:**
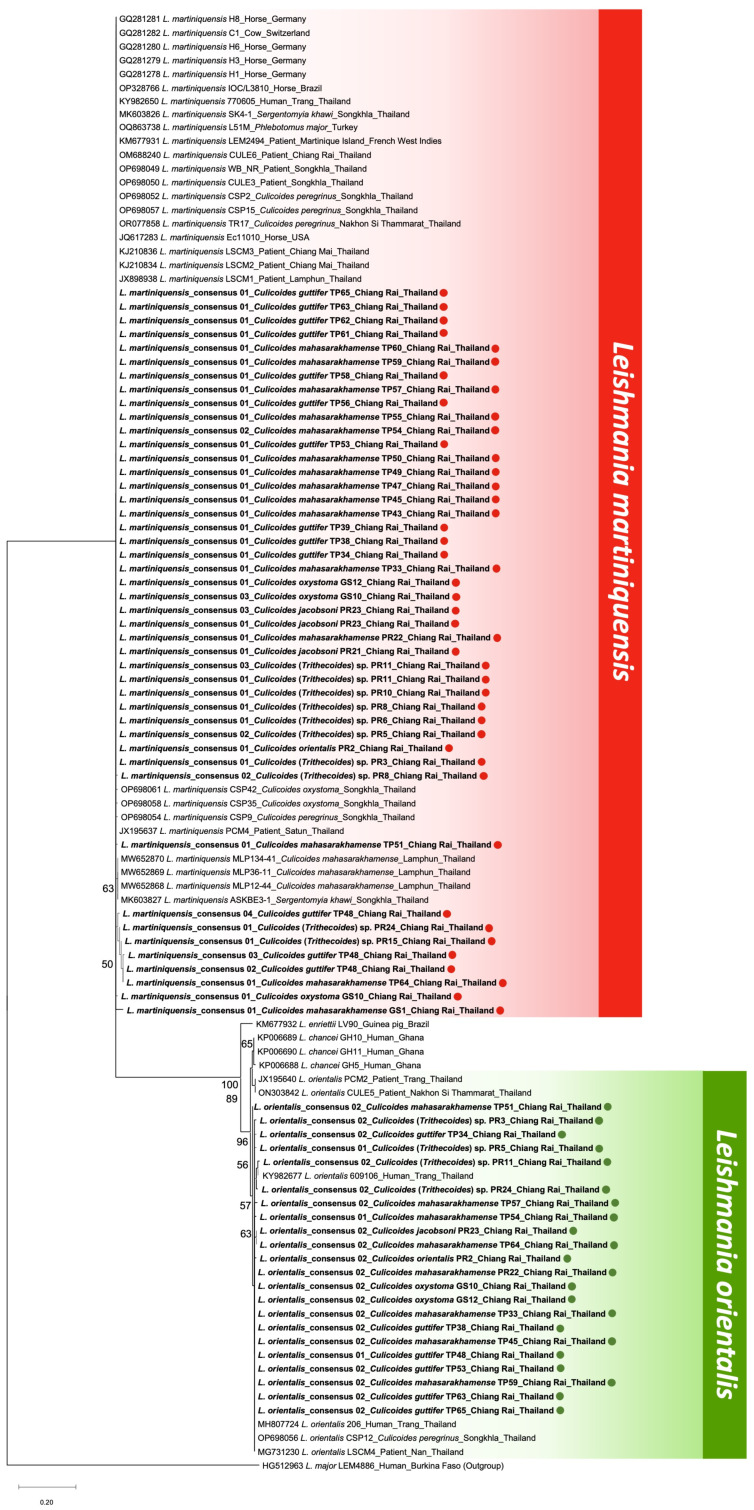
Species identification of *Leishmania* consensus sequences retrieved from *Culicoides* biting midges in this study, based on the *ITS1* phylogeny using the maximum likelihood method with the K2+G model. The colored circles indicate our sequences, including *L. martiniquensis* (red) and *L. orientalis* (green) from six *Culicoides* species. *L. major* was used as the outgroup.

The highly accurate consensus sequences obtained were aligned against the GenBank references by the BLASTn for taxonomic identification. Forty-five consensus sequences obtained from all thirty-nine *Leishmania*-positive samples were genetically identical to the *ITS1* references of *L. martiniquensis,* with high similarity percentages ranging from 97.15 to 100%. The other 22 consensus sequences retrieved from 22 *Leishmania*-positive samples matched those of *L. orientalis* references, with identity scores of 98.54–100%, as detailed in Table 1. Other *Leishmania* species were not detected in our *Culicoides* collection. Similarly, the maximum likelihood phylogenetic tree of our total consensus sequences and those *Leishmania* (*Mundinia*) references from GenBank also clearly demonstrated that our identified consensus sequences were grouped in monophyletic clades with their conspecific references, exhibiting a clear divergence between clusters of *L. martiniquensis* and *L. orientalis,* as depicted in Figure 3.

### 3.4. Genetic Diversity and Neutrality Test of Leishmania ITS1 Haplotypes

**Figure 4:**
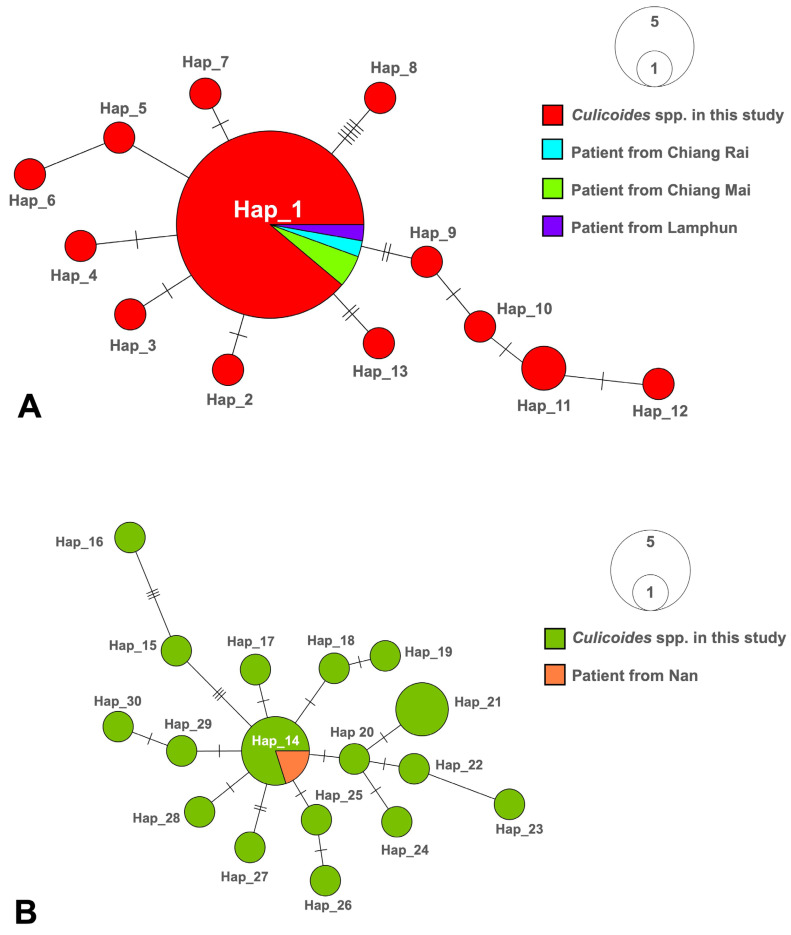
Haplotype diversity for the *ITS1* sequences of *L. martiniquensis* (**A**) and *L. orientalis* (**B**) amplified from field-caught *Culicoides* biting midges in the present study and leishmaniasis patients previously reported from Northern Thailand. The circle size proportionally represents the frequency of each unique haplotype. Hatch marks across connecting lines represent the number of nucleotide substitutions between haplotypes.

For *L. martiniquensis*, forty-five *ITS1* consensus sequences from our *Culicoides* collection in this study and those four from visceral leishmaniasis patients previously reported from Chiang Rai, Chiang Mai, and Lamphun Provinces, Northern Thailand were enrolled for haplotype network analysis. Thirteen distinct haplotypes with eighteen polymorphic sites were identified from all *L. martiniquensis* consensus sequences amplified in this investigation. As depicted in Figure 4A, Hap_1 was the most common haplotype, with thirty-six sequences (73.5%), followed by Hap_11 with two sequences (4.1%), and the remaining eleven singleton haplotypes (Hap_2 to Hap_10, Hap_12, and Hap_13).

Similarly, twenty-two *L. orientalis ITS1* consensus sequences in this study and one *ITS1* sequence from a cutaneous leishmaniasis patient previously reported from Nan Province, Northern Thailand were recruited. These consensus sequences of *L. orientalis* were divided into 17 unique haplotypes with 24 polymorphic sites. As illustrated in Figure 4B, Hap_14 with five sequences (21.7%) was the most dominant haplotype, followed by Hap_21 with three sequences (13%), and the remaining fifteen singleton haplotypes (Hap_15 to Hap_20 and Hap_22 to Hap_30).

Notably, the haplotype diversity (Hd) values for studied populations of *L. martiniquensis* and *L. orientalis* account for 0.4634 and 0.9489, respectively. On the contrary, nucleotide diversity (π) values were significantly lower for these two populations, accounting for 0.0041 and 0.0059, respectively. Furthermore, the neutrality tests were calculated as shown in Table 2, indicating that the values of Tajima’s *D*, as well as Fu and Li’s *D*, for *L. martiniquensis* (−2.3065 and −3.0583, respectively) and *L. orientalis* (−2.8127 and −2.6740, respectively) were significantly negative. All *ITS1* haplotypes with associated GenBank accession numbers and hosts are detailed in Table S2.

## 4. Discussion

The incidence of non-imported leishmaniasis caused by *Leishmania* (*Mundinia*) has continuously been increasing in Northern Thailand over recent years, raising public health awareness to prevent and control the transmission of this emerging vector-borne disease [11,12,22]. Although it has formerly been known that phlebotomine sand flies were the natural vectors of *Leishmania* worldwide, recent experimental studies demonstrated the successful colonization of metacyclic *Mundinia* promastigotes occurring in *Culicoides*, but neither in *Phlebotomus* nor in *Lutzomyia* sand flies [23,24,25]. Additionally, previous publications indicated that the *Leishmania* prevalence rates in sand fly species caught from endemic areas in Northern and Southern Thailand are relatively low [17,18,19,21,22,42]. These findings therefore implicate *Culicoides* biting midges as the most likely principal vectors responsible for autochthonous transmission of these *Mundinia* parasites.

In this work, we comparatively investigate *Culicoides* diversity and *Leishmania* prevalence in the affected village and Tham Phra cave, which are both in the same area. With special attention, we selected Tham Phra cave as one of our midge collection localities, since it is both a place of religious worship and a tourist destination that locals and visitors from abroad frequently visit throughout the year. As previously reported, cave visitors may face an increased risk of zoonotic vector-borne infections, including leishmaniasis, potentially transmitted by cave-dwelling hematophagous insects [43,44]. Accordingly, the findings from our investigation will help fill the knowledge gap regarding the diversity, species richness, and transmission dynamics of cave-dwelling insects in Northern Thailand, which remain far from comprehensive.

It was found that the *Culicoides* collection from the patient’s residence had higher species richness than other collections from the cave and the grocery store. This greater diversity might be explained by the fact that the patient’s house was adjacent to a forest edge, where certain types of soil and other environmental factors possibly favor the breeding, oviposition, and larval development of *Culicoides* species [45,46,47]. In the forest edge, the availability of blood meal sources from peridomestic animals and wildlife and low wind speed, which will not disperse midges away from the traps, are also important factors contributing to the increased diversity and abundance of *Culicoides* species [48,49,50,51]. This study also represents preliminary data identifying two common *Meijerehelea* species—namely *C. guttifer* and *C. mahasarakhamense*—as cave dwellers in Northern Thailand. However, these two species are not cave-specific and have also been reported to be widespread in various ecological habitats, i.e., villages, agricultural areas, and forest edges, throughout Thailand [48].

Based on the *ITS1*-PCR findings, six *Culicoides* species—namely *C. mahasarakhamense*, *C. guttifer*, *C.* (*Trithecoides*) spp., *C. jacobsoni*, *C. oxystoma*, and *C. orientalis*—tested positive for *Leishmania,* with an overall infection prevalence of 26.7%. Of concern is that a high prevalence of *Leishmania* infection (34.8%) was observed in cavernicolous specimens, indicating an increased risk of parasite exposure in the cave. Our results are also consistent with a prior study that found a high infection prevalence of *Leishmania* (21.2%) in three midge species, including *C. peregrinus*, *C. oxystoma*, and *C. mahasarakhamense*, collected from the active transmission area in Songkhla Province, Southern Thailand [52]. Of interest is that *Leishmania* was mostly detected with high frequency in the predominant *Culicoides* species, including *C.* (*Trithecoides*) spp. from the patient’s house as well as *C. guttifer* and *C. mahasarakhamense* from the cave. These findings corroborate previous studies that proposed that the suspected vector is more likely to be the most common species because of its dominance, which presumably increases the likelihood of disease transmission [45,52].

Apart from *C. mahasarakhamense,* which has formerly been reported as a putative leishmaniasis vector [31], our study represents the first molecular evidence of *Leishmania* in *C. guttifer*, *C.* (*Trithecoides*) spp., *C. jacobsoni*, *C. oxystoma*, and *C. orientalis* in Northern Thailand. However, these suspected vectors have been known to be livestock-associated species that mainly feed on peridomestic animals [48,52,53,54,55,56]. This implies that autochthonous *Leishmania* parasites circulate in animal reservoirs, and the most likely mode of transmission is therefore zoonotic rather than anthroponotic. Unfortunately, no blood-engorged specimens were present in our midge collection. To our knowledge, only three *Culicoides* species in Thailand—namely *C. brevitarsis* [48], *C. imicola* [54], and *C. oxystoma* [54]—have thus far been recorded for their opportunistic behavior of human feeding, implicating them for potential participation in pathogen transmission to humans. Therefore, host preference analysis of blood-engorged specimens, particularly of prevalent midge species, from leishmaniasis-affected sites is further required to determine which vector species is mainly responsible for the transmission of human leishmaniasis in our investigation area.

Given the high sensitivity and specificity, the *ITS1*-PCR has been recommended as a useful method in combination with Sanger sequencing for molecular diagnosis and epidemiological studies of leishmaniasis, particularly in endemic areas [52,57,58]. Additionally, the *ITS1* region has previously been used to demonstrate intraspecific genetic variability and phylogeographic distribution patterns in several *Leishmania* species, including *L*. (*L*.) *donovani* complex [59,60], *L*. (*L*.) *major* [61], and *L*. (*L*.) *tropica* [62]. Due to its high sequence accuracy, Sanger sequencing remains the gold standard for molecular species identification. However, for mixed *ITS1* amplicons originating from multiple *Leishmania* species, the identification cannot rely on the Sanger sequencing because this method generates a single sequence that represents only a single species, resulting in low taxonomic resolution. In this work, we speculated that individuals of *Culicoides* may harbor co-infections with more than one *Leishmania* species. For this reason, the nanopore-based metabarcoding approach was applied to produce high throughput data of *ITS1* amplicon sequences, providing us with highly accurate taxonomic assignments and enabling genetic variation analysis.

With high phylogenetic resolution, *L. martiniquensis* was ubiquitously identified in all PCR-positive specimens of six midge species, and more than half of these positives were also co-infected with *L. orientalis*. This suggests that these parasites share midge vector species and circulate sympatrically in the same transmission area. Our findings differ from those of our earlier study conducted in Songkhla Province, Southern Thailand, which revealed a low prevalence of co-infection just in two individuals of *C. perigrenus* and *C. oxystoma* [52]. The low prevalence ratio in our previous study may have been due to the limited number of plasmid clones with insert sequenced by the Sanger method, possibly leading to an underestimation of parasite abundance.

This study also provides preliminary data on the intraspecific genetic diversity of these *Mundinia* parasites. The extremely low degree of nucleotide diversity in the *ITS1* region was noted in two parasite populations, which might be attributed to their small effective population size. Nevertheless, it seems that the intraspecific variability of this genetic marker can distinguish genetic divergence within these two populations with higher haplotype diversity. Due to having a considerably higher haplotype diversity index and more unique haplotypes, the *L. orientalis* population exhibited more genetic diversity than that of *L. martiniquensis*. Our results are also consistent with the previous study that revealed a significantly high genetic diversity of *L. orientalis* isolates among asymptomatic HIV-positive people residing in Chiang Rai Province [58].

Higher haplotype diversity and relatively low nucleotide diversity were observed in these two parasite populations, suggesting that there had been a recent divergence in the population [63]. Consistently, their haplotype networks show a star-like distribution, exhibiting a numerical dominating central haplotype surrounded by multiple descendant haplotypes with only one or two base differences [64]. This star-like network pattern suggests that descendant haplotypes have recently originated from the dominant haplotype, indicating a population expansion. Significantly negative neutrality statistics substantiate this finding, indicating rapid population growth or a selective sweep [65,66]. More importantly, the occurrence of such novel haplotypes denotes an evolutionary process of parasites to adapt to a variety of hosts and biological niches across various geographic localities.

Interestingly, three collection sites in this study shared dominant haplotypes, namely Hap_1 for *L. martiniquensis* and Hap_14 for *L. orientalis* in several infected *Culicoides* species, indicating possible gene flow across the sites. This further implies the existence of animal reservoirs that serve as blood sources for a variety of infected midge species, hence maintaining the frequency of the dominant haplotype [67]. More importantly, dominant haplotypes of both *Mundinia* species were perfectly identical to those identified in all patients previously reported in northern provinces of Thailand [5,6,12,68], strongly supporting the vectorial role of *Culicoides* biting midges in the autochthonous transmission of leishmaniasis in Northern Thailand.

Essentially, these results unveiled, for the first time, the transmission dynamics and genetic diversity of two *Leishmania* (*Mundinia*) parasites co-circulating among *Culicoides* species in the endemic area of leishmaniasis in Northern Thailand. Accordingly, further entomological studies of host preference analysis and the investigation of animal reservoirs in several affected localities are required to deeply comprehend the transmission biology of this neglected disease, which would facilitate the development of vector control strategies effectively.

## 5. Conclusions

In this paper, we successfully applied nanopore sequencing technology to assess the infection prevalence and regional genetic diversity of *L. martiniquensis* and *L. orientalis* in *Culicoides* biting midges collected from the affected human settlements and Tham Phra cave in Chiang Rai Province, Northern Thailand. *L. martiniquensis* and *L. orientalis* sympatrically circulate in several midge species with a high infection prevalence across these localities. Besides *C. mahasarakhamense* previously recorded, our findings implicate, for the first time, several *Culicoides* species—namely *C. guttifer*, *C.* (*Trithecoides*) spp., *C. jacobsoni*, *C. oxystoma*, and *C. orientalis*—as new putative vectors of leishmaniasis in Northern Thailand. The genetic diversity analysis also revealed that these two parasite species recently underwent population divergence. The dominant haplotype of each species was perfectly identical to those identified in all symptomatic patients from Northern Thailand, substantiating the pivotal role of *Culicoides* midges in disease transmission. Ultimately, significant data from this research will be useful in assessing the *Leishmania* infection risk and developing vector control strategies to effectively mitigate the transmission of this neglected disease, particularly in endemic regions of Northern Thailand.

## Figures and Tables

**Table 1:** Species composition of *Culicoides* biting midges with associated infection prevalence and molecular identification of *Leishmania* parasites using MinION^®^ nanopore sequencing across three collection sites in the present study.

Collection Sites	Genus (Subgenus/Species Group)	Species	No. Males	No. Non-Engorged Females		No. Females with Parasites Detected	Midge Specimen Vouchers with Parasite Identification	
				Parous	Gravid		*L. martiniquensis*	*L. orientalis*
Patient Residence Mae Kon, Muang District Chiang Rai Province (19°51′05.8″ N, 99°39′37.1″ E)	*C.* (*Hoffmania*)	*insignipennis*	2	15				
	*C.* (*Hoffmania*)	*malayae*		6				
	*C.* (*Hoffmania*)	*liui*		5				
	*C.* (*Hoffmania*)	*innoxius*		5				
	*C.* (*Hoffmania*)	*sumatrae*		2				
	*C.* (*Trithecoides*)	spp.	5	17	1	8	PR3, PR5, PR6, PR8, PR10, PR11, PR15, PR24	PR3, PR5, PR11, PR24
	*C.* (*Avaritia*)	*orientalis*		12		1	PR2	PR2
	*C.* (*Avaritia*)	*jacobsoni*		4		2	PR21, PR23	PR23
	*C.* (*Remmia*)	*oxystoma*			2			
	*C.* (*Shermani* group)	*thurmanae*		1				
	*C.* (*Meijerehelea*)	*mahasarakhamense*			1	1	PR22	PR22
		Total	7	67	4	12/71 (16.9%)	12/71 (16.9%)	7/71 (9.9%)
Grocery Shop Mae Kon, Muang District Chiang Rai Province (19°50′56.3″ N, 99°39′50.1″ E)	*C.* (*Remmia*)	*oxystoma*		3		2	GS10, GS12	GS10, GS12
	*C.* (*Avaritia*)	*jacobsoni*		2				
	*C.* (*Meijerehelea*)	*mahasarakhamense*		1		1	GS1	
		Total		6		3/6 (50%)	3/6 (50%)	2/6 (33.3%)
Tham Phra Cave Mae Yao, Muang District Chiang Rai Province (19°55′03.5″ N, 99°47′19.5″ E)	*C.* (*Meijerehelea*)	*guttifer*	7	34	7	11	TP34, TP38, TP39, TP48, TP53, TP56, TP58, TP61, TP62, TP63, TP65	TP34, TP38, TP48, TP53, TP63, TP65
	*C.* (*Meijerehelea*)	*mahasarakhamense*	2	16	12	13	TP33, TP43, TP45, TP47, TP49, TP50, TP51, TP54, TP55, TP57, TP59, TP60, TP64	TP33, TP45, TP51, TP54, TP57, TP59, TP64
		Total	9	50	19	24/69 (34.8%)	24/69 (34.8%)	13/69 (18.8%)
Grand total			16	146		39/146 (26.7%)	39/146 (26.7%)	22/146 (15.1%)

**Table 2:** Genetic diversity and neutrality statistics of *Leishmania ITS1* haplotypes identified in field-caught *Culicoides* biting midges in this study and in patients previously reported from Northern Thailand.

Parasite Population	Total Sample Size	No. Haplotypes (H)	No. Polymorphic Sites (S)	Average no. of Nucleotide Differences (k)	Haplotype Diversity (Hd) ± SD	Nucleotide Diversity (π) ± SD	Tajima’s *D*	Fu and Li’s *D*
*L. martiniquensis* from *Culicoides* (45) and patients (4)	49	13	18	3.2179	0.4634 ± 0.0903	0.0041 ± 0.0031	−2.3065 *	−3.0583 *
*L. orientalis* from *Culicoides* (22) and one patient (1)	23	17	24	2.1618	0.9489 ± 0.0326	0.0059 ± 0.0041	−2.8127 **	−2.6740 *

* *p*-value < 0.05. ** *p*-value < 0.01.

## Data Availability

All data generated or analyzed during this study are included in this published article and its supplementary information files.

## Supplementary Materials

The following supporting information can be downloaded at: https://www.mdpi.com/article/10.3390/insects15050327/s1, Table S1: Statistics of nanopore sequenced reads and BLASTn identification. Table S2: Haplotypes of 49 *L. martiniquensis* and 23 *L. orientalis ITS1* sequences amplified from *Culicoides* biting midges collected in this study and patients previously reported from Northern Thailand.
