# Supplementary material for: Nanopore-Based Surveillance of Leishmania Parasites in Culicoides Latrielle (Diptera: Ceratopogonidae) Caught from the Affected Community and Tham Phra Cave in Chiang Rai Province, the Endemic Area of Leishmaniasis in Northern Thailand
Source: Insects. 2024 May 2;15(5):327. doi: 10.3390/insects15050327 (PMC11122411; doi:10.3390/insects15050327)
Supplement: Supplementary file 1 [file insects-15-00327-s001.zip › insects-2981883-supplementary.pdf]

Table S1. Statistics of nanopore sequenced reads and BLASTn identification.

Species	Code	Consensus	No. reads	Length (bp)	BLASTn Identification	% identity
C. orientalis	PR2	Consensus_01	14,131	281	*L. martiniquensis* 770605 (KY982650)	100
		Consensus_02	2,558	272	*L. orientalis* 378 (KY982674)	99.63
C. (*Trithecoides*) sp.	PR3	Consensus_01	14,037	281	*L. martiniquensis* 770605 (KY982650)	100
		Consensus_02	4,662	270	*L. orientalis* 378 (KY982674)	98.89
C. (*Trithecoides*) sp.	PR5	Consensus_01	2,239	273	*L. orientalis* 340 (KY982673)	99.63
		Consensus_02	479	320	*L. martiniquensis* 770605 (KY982650)	100
C. (*Trithecoides*) sp.	PR6	Consensus_01	15,242	281	*L. martiniquensis* 770605 (KY982650)	100
C. (*Trithecoides*) sp.	PR8	Consensus_01	17,204	281	*L. martiniquensis* 770605 (KY982650)	100
		Consensus_02	3,561	306	*L. martiniquensis* 770605 (KY982650)	99.64
C. (*Trithecoides*) sp.	PR10	Consensus_01	23,000	279	*L. martiniquensis* SK4-1 (MK603826)	99.64
C. (*Trithecoides*) sp.	PR11	Consensus_01	13,364	281	*L. martiniquensis* 770605 (KY982650)	100
		Consensus_02	3,747	273	*L. orientalis* PCM2 (JX195640)	99.26
		Consensus_03	1,836	313	*L. martiniquensis* 770605 (KY982650)	99.64
C. (*Trithecoides*) sp.	PR15	Consensus_01	17,343	279	*L. martiniquensis* SK4-1 (MK603826)	98.92
C. jacobsoni	PR21	Consensus_01	15,408	281	*L. martiniquensis* 770605 (KY982650)	100
C. mahasarakhamense	PR22	Consensus_01	15,462	281	*L. martiniquensis* 770605 (KY982650)	100
		Consensus_02	5,228	273	*L. orientalis* 340 (KY982673)	100
C. jacobsoni	PR23	Consensus_01	14,569	280	*L. martiniquensis* 770605 (KY982650)	99.64
		Consensus_02	2,235	273	*L. orientalis* 340 (KY982673)	99.63
		Consensus_03	1,645	313	*L. martiniquensis* 770605 (KY982650)	100
C. (*Trithecoides*) sp.	PR24	Consensus_01	14,232	282	*L. martiniquensis* 770605 (KY982650)	99.29
		Consensus_02	2,428	275	*L. orientalis* 609106 (KY982677)	99.27
C. mahasarakhamense	GS1	Consensus_01	18,970	280	*L. martiniquensis* 770605 (KY982650)	97.15
C. oxystoma	GS10	Consensus_01	8,516	281	*L. martiniquensis* 770605 (KY982650)	99.64
		Consensus_02	6,043	273	*L. orientalis* 609106 (KY982677)	98.54
		Consensus_03	1,512	312	*L. martiniquensis* 770605 (KY982650)	100
C. oxystoma	GS12	Consensus_01	13,130	281	*L. martiniquensis* 770605 (KY982650)	100
		Consensus_02	1,484	273	*L. orientalis* 409537 (KY982675)	100
C. mahasarakhamense	TP33	Consensus_01	12,061	281	*L. martiniquensis* 770605 (KY982650)	100
		Consensus_02	3,734	271	*L. orientalis* 409537 (KY982675)	99.26
C. guttifer	TP34	Consensus_01	14,131	281	*L. martiniquensis* 770605 (KY982650)	100
		Consensus_02	862	273	*L. orientalis* 409537 (KY982675)	100
C. guttifer	TP38	Consensus_01	13,894	281	*L. martiniquensis* 770605 (KY982650)	100
		Consensus_02	790	272	*L. orientalis* 378 (KY982674)	100

Table S1. Statistics of nanopore sequenced reads and BLASTn identification. (Continued)

Species	Code	Consensus	No. reads	Length (bp)	BLASTn Identification	% identity
C. guttifer	TP39	Consensus_01	14,850	281	*L. martiniquensis* 770605 (KY982650)	100
C. mahasarakhamense	TP43	Consensus_01	13,242	281	*L. martiniquensis* 770605 (KY982650)	100
C. mahasarakhamense	TP45	Consensus_01	16,963	281	*L. martiniquensis* 770605 (KY982650)	100
		Consensus_02	2,381	273	*L. orientalis* 409537 (KY982675)	100
C. mahasarakhamense	TP47	Consensus_01	10,410	281	*L. martiniquensis* 770605 (KY982650)	100
C. guttifer	TP48	Consensus_01	1,298	272	*L. orientalis* 378 (KY982674)	100
		Consensus_02	12,317	279	*L. martiniquensis* SK4-1 (MK603826)	98.56
		Consensus_03	3,673	309	*L. martiniquensis* SK4-1 (MK603826)	98.92
		Consensus_04	2,575	309	*L. martiniquensis* SK4-1 (MK603826)	99.28
C. mahasarakhamense	TP49	Consensus_01	16,714	281	*L. martiniquensis* 770605 (KY982650)	100
C. mahasarakhamense	TP50	Consensus_01	17,396	281	*L. martiniquensis* 770605 (KY982650)	100
C. mahasarakhamense	TP51	Consensus_01	13,551	281	*L. martiniquensis* 770605 (KY982650)	100
		Consensus_02	3,257	275	*L. orientalis* 272078 (KY982669)	99.64
C. guttifer	TP53	Consensus_01	13,509	281	*L. martiniquensis* 770605 (KY982650)	100
		Consensus_02	3,265	273	*L. orientalis* 409537 (KY982675)	100
C. mahasarakhamense	TP54	Consensus_01	1,319	273	*L. orientalis* 378 (KY982674)	100
		Consensus_02	16,190	281	*L. martiniquensis* 770605 (KY982650)	100
C. mahasarakhamense	TP55	Consensus_01	14,892	281	*L. martiniquensis* 770605 (KY982650)	100
C. guttifer	TP56	Consensus_01	16,861	281	*L. martiniquensis* 770605 (KY982650)	100
C. mahasarakhamense	TP57	Consensus_01	14,464	281	*L. martiniquensis* 770605 (KY982650)	100
		Consensus_02	1,189	272	*L. orientalis* 378 (KY982674)	99
C. guttifer	TP58	Consensus_01	13,718	281	*L. martiniquensis* 770605 (KY982650)	100
C. mahasarakhamense	TP59	Consensus_01	12,848	281	*L. martiniquensis* 770605 (KY982650)	100
		Consensus_02	1,802	273	*L. orientalis* 409537 (KY982675)	100
C. mahasarakhamense	TP60	Consensus_01	11,610	281	*L. martiniquensis* 770605 (KY982650)	100
C. guttifer	TP61	Consensus_01	14,104	281	*L. martiniquensis* 770605 (KY982650)	100
C. guttifer	TP62	Consensus_01	15,557	281	*L. martiniquensis* 770605 (KY982650)	100
C. guttifer	TP63	Consensus_01	20,660	281	*L. martiniquensis* 770605 (KY982650)	100
		Consensus_02	3,804	272	*L. orientalis* 378 (KY982674)	100
C. mahasarakhamense	TP64	Consensus_01	19,052	279	*L. martiniquensis* SK4-1 (MK603826)	98.56
		Consensus_02	1,197	273	*L. orientalis* 409537 (KY982675)	99.26
C. guttifer	TP65	Consensus_01	12,702	281	*L. martiniquensis* 770605 (KY982650)	100
		Consensus_02	2,541	272	*L. orientalis* 409537 (KY982675)	99.63

Table S2. Haplotypes of 49 *L. martiniquensis* and 23 *L. orientalis* *ITS1* sequences amplified from *Culicoides* biting midges collected in this study and patients previously reported from Northern Thailand.

Species	Haplotypes (frequency)	Length (bp)	Accession numbers of associated *Leishmania ITS1* sequences and hosts
L. martiniquensis	Hap_1 (36)	259	PP586221 (*C. orientalis*_PR2; *C.* (*Trithecoides*) sp._PR3, PR5, PR6, PR8, PR11; *C. jacobsoni*_PR21, PR23; *C. oxystoma*_GS10, GS12; *C. mahasarakhamense*_PR22, TP33, TP43, TP45, TP47, TP49, TP50, TP54, TP55, TP57, TP59, TP60; *C. guttifer*_TP34, TP38, TP39, TP53, TP56, TP58, TP61, TP62, TP63, TP65), OM688240 (patient from Chiang Rai), JX898938 (a patient from Lamphun), KJ210834, and KJ210836 (two patients from Chiang Mai)
	Hap_2 (1)	259	PP586222 (*C. oxystoma*_GS10)
	Hap_3 (1)	259	PP586223 (*C.* (*Trithecoides*) sp._PR08)
	Hap_4 (1)	257	PP586224 (*C.* (*Trithecoides*) sp._PR10)
	Hap_5 (1)	258	PP586225 (*C. jacobsoni*_PR23)
	Hap_6 (1)	259	PP586226 (*C.* (*Trithecoides*) sp._PR11)
	Hap_7 (1)	259	PP586227 (*C. mahasarakhamense*_TP51)
	Hap_8 (1)	257	PP586228 (*C. mahasarakhamense*_GS1)
	Hap_9 (1)	259	PP586229 (*C.* (*Trithecoides*) sp._PR24)
	Hap_10 (1)	257	PP586230 (*C.* (*Trithecoides*) sp._PR15)
	Hap_11 (2)	257	PP586231 (*C. guttifer*_TP48; *C. mahasarakhamense*_TP64)
	Hap_12 (1)	257	PP586232 (*C. guttifer*_TP48)
	Hap_13 (1)	257	PP586233 (*C. guttifer*_TP48)
L. orientalis	Hap_14 (5)	251	PP586234 (*C. mahasarakhamense*_PR22, TP45; *C. oxystoma*_GS12; *C. guttifer*_TP53) and MG731230 (a patient from Nan)
	Hap_15 (1)	251	PP586235 (C. (*Trithecoides*) sp._PR11)
	Hap_16 (1)	253	PP586236 (C. (*Trithecoides*) sp._PR24)

Table S2. Haplotypes of 49 *L. martiniquensis* and 23 *L. orientalis ITS1* sequences amplified from *Culicoides* biting midges collected in this study and patients previously reported from Northern Thailand. (Continued)

Species	Haplotypes (frequency)	Length (bp)	Accession numbers of associated *Leishmania ITS1* sequences and hosts
	Hap_17 (1)	251	PP586237 (*C.* (*Trithecoides*) sp._PR5)
	Hap_18 (1)	248	PP586238 (*C.* (*Trithecoides*) sp._PR3)
	Hap_19 (1)	250	PP586239 (*C. mahasarakhamense*_TP54)
	Hap_20 (1)	250	PP586240 (*C. orientalis*_PR2)
	Hap_21 (3)	250	PP586241 (*C. guttifer*_TP38, TP48, TP63)
	Hap_22 (1)	249	PP586242 (*C. mahasarakhamense*_TP33)
	Hap_23 (1)	251	PP586243 (*C. mahasarakhamense*_TP59)
	Hap_24 (1)	251	PP586244 (*C. oxystoma*_GS10)
	Hap_25 (1)	250	PP586245 (*C. guttifer*_TP65)
	Hap_26 (1)	253	PP586246 (*C. mahasarakhamense*_TP51)
	Hap_27 (1)	250	PP586247 (*C. mahasarakhamense*_TP57)
	Hap_28 (1)	251	PP586248 (*C. guttifer*_TP34)
	Hap_29 (1)	251	PP586249 (*C. jacobsoni*_PR23)
	Hap_30 (1)	251	PP586250 (*C. mahasarakhamense*_TP64)
